# Adverse drug reactions in primary care: a scoping review

**DOI:** 10.1186/s12913-019-4651-7

**Published:** 2020-01-06

**Authors:** H. Khalil, C. Huang

**Affiliations:** 1 School of Psychology and Public Health, Department of Public Health, Latrobe University, Collins Street., Melbourne, Vic 3000 Australia; 2 Monash University, Clayton, Vic 3825 Australia

## Abstract

**Background:**

Medication-related adverse events, or adverse drug reactions (ADRs) are harmful events caused by medication. ADRs could have profound effects on the patients’ quality of life, as well as creating an increased burden on the healthcare system. ADRs are one of the rising causes of morbidity and mortality internationally, and will continue to be a significant public health issue with the increased complexity in medication, to treat various diseases in an aging society. This scoping review aims to provide a detailed map of the most common adverse drug reactions experienced in primary healthcare setting, the drug classes that are most commonly associated with different levels/types of adverse drug reactions, causes of ADRs, their prevalence and consequences of experiencing ADRs.

**Methods:**

We systematically reviewed electronic databases Ovid MEDLINE, Embase, CINAHL Plus, Cochrane Central Register of Controlled Trials, PsycINFO and Scopus. In addition, the National Patient Safety Foundation Bibliography and the Agency for Health Care Research and Quality and Patient Safety Net Bibliography were searched. Studies published from 1990 onwards until December 7, 2018 were included as the incidence of reporting drug reactions were not prevalent before 1990. We only include studies published in English.

**Results:**

The final search yielded a total of 19 citations for inclusion published over a 15-year period that primarily focused on investigating the different types of adverse drug reactions in primary healthcare. The most causes of adverse events were related to drug related and allergies. Idiosyncratic adverse reactions were not very commonly reported. The most common adverse drug reactions reported in the studies included in this review were those that are associated with the central nervous system, gastrointestinal system and cardiovascular system. Several classes of medications were reported to be associated with adverse events.

**Conclusion:**

This scoping review identified that the most causes of ADRs were drug related and due to allergies. Idiosyncratic adverse reactions were not very commonly reported in the literature. This is mainly because it is hard to predict and these reactions are not associated with drug doses or routes of administration. The most common ADRs reported in the studies included in this review were those that are associated with the central nervous system, gastrointestinal system and cardiovascular system. Several classes of medications were reported to be associated with ADRs.

## Background

Medication-related adverse events, or adverse drug reactions (ADRs) are harmful events caused by medication. Adverse drug reactions (ADRs) are defined by the World Health Organization (WHO) as “a response to a medication that is noxious and unintended used in man to treat” [1]. ADRs could be a result of a preventable medication error, resulting in a side-effect as a result of medication administration, or an unforeseen error such as an allergic reaction [2, 3].

ADRs could have profound effects on the patients’ quality of life, as well as creating an increased burden on the healthcare system. ADRs are one of the rising causes of morbidity and mortality internationally, and will continue to be a significant public health issue with the increased complexity in medication, to treat various diseases in an aging society. A recent study showed that ADRs accounted for approximately 3.5% of hospital admission [4, 5]. Furthermore, ADRs were the cause of ~ 197,000 deaths in Europe annually [1].

The causes and nature of adverse drug events are often complex and multifactorial. The types of adverse reactions are classified into the following categories: dose/drug related, allergic or idiosyncratic reactions. Dose-related and drug related adverse drug reactions are usually related to the dose of the medication and are usually predictable but sometimes unavoidable [6–9]. It is highly dependent on the patient’s sensitivity to the drug and combinations of medication used. It generally does not lead to severe ADR but is relatively common. An allergic drug reaction is when the patients develops an inappropriate reaction to the medication, which mostly could be avoided with a skin test prior to or through effective consultation and communication between primary care facilities and patients. An idiosyncratic adverse drug reaction is a type that is not widely understood and its severity is often quite unpredictable. This affects the fewer people and the reason for the adverse reaction may be genetically predetermined [9].

ADRs have become a significant problem in patients who are on multiple medications such as the elderly. A study has reported that as high as 75% of all aged care residents had medication discrepancies after the transition from hospital to primary care setting [6].

Most of the adverse medication events are associated with prescription errors in general practice [7]. Medication errors in general practice had a prevalence rate of 5% in England according to a large retrospective case review study [8]. With the incorporation of technology in healthcare system, the implementation of computerized prescribing systems also has a range of medication error rates that may lead to mild or severe adverse drug events [10, 11].

Another cause of adverse events is the off -label use of uncommon medications in children and patients. Off label prescribing is the process of prescribing of medications to non-approved indications by organizations such the Therapeutic Goods Administration of Australia or the Food and Drug Administration agency in the United States. Medication error or dosage error can occur in these circumstances due to the lack of evidence to support their use in non-approved conditions [12–14].

To date, there is limited data and evidence on the epidemiology of ADRs. After a preliminary search of literature, (i.e. *The Cochrane Library, JBI Database of Systematic Reviews and Implementation Reports, Ovid MEDLINE*) there are no systematic reviews, meta-analysis or scoping reviews that provide a comprehensive overview of the types of adverse events in primary care. Most of the studies available were relatively small, and often confined to individual units. Alternately, most of the current reviews focused on the occurrence of medication errors, specific interventions to reduce medication errors and medication management [15–22]. While there are several reviews on medication programs focusing on the effect of medication errors and effectiveness of interventions, they do not describe the types of adverse events [21, 22]. The review by Khalil et al., 2017 examined the effectiveness of various types of medication safety interventions to reduce mortality, emergency visits and hospital admissions. The authors found little evidence to support the benefits of organizational, professional and structural interventions addressing medication errors due to the heterogeneity of the included studies [21]. .Assiri et al., 2018 examined the prevalence of medications errors and adverse events associated with errors and risk factors associated with them. They found inconsistencies in the definitions of medications errors, methodologies used to detect adverse events and different outcome measures.

Therefore, this review sought to address the type of ADRs, the major drug classes associated with the reactions, causes of ADRs, their prevalence as well as consequences of experiencing ADRs to reduce the risk of adverse drug events in primary care. This will enable clinicians to be more informed of the adverse events and which class of drugs are associated with them. Targeted educational interventions addressing these gaps have the potential to improve patient safety. This scoping review will also be useful for researchers and healthcare providers as well as policy makers in the development of interventions to reduce adverse drug reactions in today’s primary care.

### Inclusion criteria

#### Participants

This review considered participants of any age and any condition treated and/or managed from any primary care services.

#### Concept

The concept of interest for the scoping review was the type of adverse drug reactions experienced by patients and the classes of medications associated with these adverse drug events.

#### Context

The context of the review was the primary care setting. These include; primary health care organizations, general practitioner clinics, pharmacies, outpatient clinics and any other clinics that do not classify patients as inpatients. We only excluded hospital patients.

### Types of studies

This scoping review considered quantitative study designs including experimental, descriptive and observational studies reporting any quantitative data that can be included in the review. Qualitative studies were not considered in this review as the data extracted were not eligible for inclusion as mentioned in the scoping protocol [23]. Due to time constraints, only data published in English were considered for the review. No gray literature was searched as we are interested in studies that are published in peer reviewed journals based on scientific methods that use evidence to develop conclusions.

### Search strategy

The search strategy aimed to identify studies published from 1990 to 2018. A three-step search strategy was utilized in this review. An initial limited search of Ovid MEDLINE, JBI Database of Systematic Reviews and Implementation Reports and Cochrane Central Register of Controlled Trials was undertaken followed by analysis of the text words contained in the title and abstract and of the index terms used to describe the article. A second search using all identified keywords and index terms was undertaken across all included databases. The following databases were searched on December 7, 2018: Ovid MEDLINE, Embase, CINAHL Plus, Cochrane Central Register of Controlled Trials, PsycINFO and Scopus*.* The search strategy of all the databases followed the same strategy shown in Appendix I. In addition, the National Patient Safety Foundation Bibliography and the Agency for Health Care Research and Quality and Patient Safety Net Bibliography were searched. Studies published from 1990 onwards until December 7, 2018 were included as the incidence of reporting drug reactions were not prevalent before 1990. The reference list of all identified reports and articles were searched for additional studies. The following keywords were used: patient safety, adverse events, harmful incidents, primary care, aged care, ambulatory care, general practice and home healthcare. These were used along with a comprehensive list of variations of these key terms.

## Method

### Data extraction

Relevant data were extracted from the included studies to address the review question using the methodology outlined by Peters et al. [24, 25] The data extracted followed the template developed in the protocol [23]. .Please refer to the search strategy published in the protocol [23].

The data extracted included the following: author(s), year of publication, origin/country of origin (where the study was published or conducted), aims/purpose, study population, methodology/methods, context, types of adverse drug reactions experienced by patients and the classes of medications associated with them as shown in Tables 1 and 2.

**Table 1:** Characteristics of studies (Part 1)

Study author	Year of publication	Origin/ country of origin	Aims/purpose	Study population	Methodology/ methods	Context
Gandhi et al [26]	2003	USA	To determine the rates, types, severity and preventability of adverse events related to drugs and to identify preventative strategies	1202 outpatients visiting adult primary care practices in Boston (2 hospital-based and 2 community based)	Prospective cohort study involving a survey of patients and a chart review of patients visiting primary care practices	Primary care practices
Gurwitz et al [27]	2005	USA, Canada	To assess the incidence of adverse drug and risk factors of adverse drug events in the long-term care setting	Long stay residents of two large academic long-term care facilities in Connecticut (1229 beds), USA and Ontario, Canada.	Prospective cohort study involving review of medical records for possible drug related events	Long term Care facilities
Hakkarainen *et a* [28]	2014	Sweden	To estimate the prevalence of adverse drug events (ADEs) in 3 months and categorize them into preventable ADEs	5025 adults’ medical records were drawn from inpatient care, outpatient clinics and primary care departments from the Swedish county council	Retrospective review of medical records	Outpatients clinic and primary care departments
Jacobs & Ross [29]	2012	South Africa	To examine the adverse effect in a sample of outpatient in Multi-drug resistant Tuberculosis clinic	Patients with multidrug-resistant tuberculosis	A retrospective review of 350 patients with multidrug resistance tuberculosis in 2010 to 2011	Outpatient clinic
Kaushal et al [30]	2007	USA	To investigate the rates and types of adverse drug events in a pediatric ambulatory setting	Children under 21 years that received a prescription during their visits	A prospective cohort study of prescription reviews, telephone surveys at 6 office practices in Greater Boston over 2 months period	Ambulatory care
Kowski *et al* [31]	2016	Germany	To assess the adverse effects of antiepileptic drugs (AEDs) in epilepsy outpatient clinic.	Epilepsy patients taking antiepileptic.	Patients over 16 years age with epilepsy for over 12 months were asked to complete Liverpool Adverse Event Profile (LAEP).	Epilepsy outpatient
Lahon *et al* [21]	2012	India	To study the prevalence and pattern, as well as the causality, severity and preventability of adverse drug reaction (ADR) of antipsychotics, antidepressants and mood stabilizers prescribed at this particular hospital	222 patients visiting	Retrospective observational study of	Psychiatric outpatient clinic
Li & Tian [33]	2014	Singapore	To assess adverse drug reaction in oral antibiotics used in dermatological indications	28 outpatient cases from National Skin Centre, Singapore in 2013	Retrospective case review of outpatient reported on adverse drug reaction	Outpatient clinics
Lin *et al* [34]	2008	Taiwan	To identify the risk factors, prevalence, and adverse outcomes of potentially inappropriate medication use	Patients aged 65+ with long-term diseases requiring prescriptions for treatment	Observational cohort study of computerised claims of elderly patients aged 65+ with chronic conditions requiring long term prescriptions from a tertiary medical centre in March 2005	Primary care
Milligan *et al* [35]	2012	UK	To analyse adverse drug events in relation to insulin therapy/oral glucose- lowering agents in care home setting	Older patients in nursing homes.	A retrospective audit of Reports from National Patient Safety Agency between January 1st 2005 and December 31st 2009.	Residential Nursing homes
Montserrat-Capella *et al* [36]	2015	Mexico,Peru, Brazil, Colombia	To determine the frequency of adverse events and preventability of such events in selected ambulatory care sites	2080 patients in outpatient clinics across Latin American countries	Multinational observational cohort study of a random selection of 2080 patients in outpatient clinics across Latin American countries.	Ambulatory care
Rosales *et al* (abstract) [37]	2015	Spain	To outline the incidence and characteristics of moderate to severe ADR to biological agents in rheumatoid arthritis (RA) patients	Rheumatoid arthritis patients	Observational longitudinal study from 1999 to 2013 of RA patients followed up in outpatient clinic	Outpatient
Schildmeijer *et al* [38]	2018	Sweden	To investigate the origin, incidence, types and preventability of adverse events that occur in patients receiving homecare	Home care patients	Retrospective record review of healthcare records of 600 patients	Home care
Shehab *et al* [39]	2008	USA	To estimate and compare the numbers and rates of emergency department visits due to systemic antibiotics related adverse effects	outpatients visiting ambulatory care practices	Retrospective audit of the National Electronic Injury Surveillance System-Cooperative Adverse Drug Event Surveillance project (2004–2006) and outpatient prescriptions from national sample surveys of ambulatory care practices	Ambulatory care practices
Theitler *et al* (Abstract) [40]	2016	Czech Republic	To evaluate the rate of adverse event in a cohort of elderly patients with epilepsy	115 epilepsy patients aged between 60 to 90	Retrospective review of computerised databases and medical records of patients aged over 65 in epilepsy outpatient clinic from February 2012 to February 2016	Outpatient
Tomlin *et al* [41]	2012	New Zealand	To examine the adverse effect in a general practice setting	338,931 patients visiting 30 general practices	A review of electronic clinical records of 338,931 patients from 2002 to 2007 from patient management systems	General practice settings
Tsang *et al* [42]	2010	UK	To identify the rate and types of adverse events in primary care	Patients that used primary care practices in the UK	Descriptive analyses of data extracted from clinical information management systems	Primary care practices
Woods *et al* [43]	2007	USA	To investigate the epidemiology of errors and adverse events in ambulatory care	Patients who experienced adverse events in ambulatory care that led to hospitalization	A retrospective review of 14,700 hospital discharge records samples in Colorado and Utah Medical Practices	Primary care
Wucherer *et al* [44]	2017	Germany	To determine prevalence and types of drug related problems in community-dwelling residents whom are positive for dementia	Participants screened positive for dementia	Retrospective analysis of medication reviews of 446 participants by pharmacists using a comprehensive baseline assessment	General Practice

**Table 2:** Characteristics of studies (Part 2)

Study author	Types of adverse events	Classes of drugs associated with drug reactions	Incidence/prevalence	causes	Consequences
Gandhi *et al* [26]	Drug related and allergic reactions. These included: central nervous system (33%), gastrointestinal events (22%) and cardiovascular events (18%)	Selective serotonin- reupake inhibitors (18, 10%) Beta-blockers (16, 9%) Angiotensin-converting-enzyme inhibtors (15, 8%) Nonsteroidal anti-inflammatory agents (15,8%) Calcium-channel blocers (12, 7%) Penicillins (7, 4%) Oral corticosteroids (7, 4%) Nonnarcotic analgesic agents (6, 3%)	The rate of adverse events reported in this staged was 27 per 100 patients	An inappropriate choice of drugs, drug interaction drug allergy in individual patients. When physicians failed to respond to medication-related symptoms when patients failed to inform physicians about medication-related symptoms.	Fatal and life threatening 0% Serious 13% Significant 87% Preventable
Gurwitz *et al* [27]	Drug related events: Neuropsychiatric - 199 (24%) Haemorrhagic - 159 (20%) Gastrointestinal - 140 (17%) Renal/electrolytes - 80 (10%) Metabolic/endocrine - 64 (8%) Dermatological - 36 (4%) Cardiovascular 36 (4%)	- Warfarin - 121 (15%) Atypical antipsychotic agents - 92 (11%) Loop diuretics - 69 (8%) Opioids - 51 (6%) Antiplatelets - 46 (6%) ACE inhibitors - 45 (6%) Antidepressants - 43 (5%) Laxatives - 43 (5%) Benzodiazepines - 39 (5%)	There were 815 adverse drug events, of which 42% were judged preventable. The overall rate of adverse drug events were 9.8 per 100 resident- months.	Dispensing errors Monitoring errors Prescribing errors (wrong drug choice, wrong dose)	Death (< 1%) Life threatening (4%) Serious (23%) Less serious (72%)
Hakkarainen *et al* [28]	Drug related - gastrointestinal disorders (21.6%) - General disorders and administration site conditions (12.3%) - Cardiac disorders (8.9%) - Nervous system disorders (8.8%) - Vascular disorders (8.8%) - Psychiaric disorders (7.8%) - Investigations (5.8%) Respiratory, thoracic and mediastinal disorders (4.7%)	- Drugs for the nervous system (psychoanaleptics 17.8%, psycholeptics 15.6%, analgesics 14.1%) - Drugs for the cardiovascular system to 37.8% (β-adrenoceptor blocking agents 15.6%, diuretics 14.1%, agents acting on the renin-angiotensin system 11.9%). - Drugs for blood 9.6% (antithrombotic agents 8.1%) - Drugs for the musculoskeletal system 8.2% (antiinflammatory and anti-rheumatic products 7.4%)	The 3 month prevalence of serious ADEs in the general population was 1.2% (95% CI 0.9, 1.6%). - In 4970 included individuals the prevalence of ADEs was 12.0% (95% confidence interval (CI) 11.1, 12.9%).	-Advanced age -Other causes not specified	• Hospital admission (14%)
Jacobs & Ross [29]	Drug related, allergic and idiosyncratic - Seizures - Peripheral neuropathy - Hearing loss and vestibular disturbances - Psychoses and confusion - Gastrointestinal symptoms (nausea, vomiting and diarrhoea) - Jaundice - Arthralgia - Skin rashes	Highly active antiretroviral therapy and Anti-tuberculosis Drugs -terizidone, -ethionamide -Rifampicin -Ethionamide -Stavudine	Adverse events were recorded for 80.6% of patients	- HIV positive patients were more likely to experience ADR than HIV negative patients	• Unspecified
Kaushal *et al* [30]	Drug related, allergic	- Penicillin or derivative -Steroids, inhaled - Antifungal, topical - Antihistamine - Histamine H2 receptor antagonist - Bronchodilators, inhaled - Cephalosporins - Macrolides - Steroids, oral - Ophthalmic preparations - Stimulants - Ibuprofen	There was a total of 57 preventable ADEs (rate 3%; 95% CI, 3–4%) and 226 non- preventable ADEs (rate 13%; 95% CI,11–15%) in the medical care of 1788 patients.	Drug administration Lack of patient education about adverse events Delay in notifying about adverse events	None of the preventable ADEs were life threatening,8(14%) were serious
Kowski *et al* [31]	Drug related sleepiness, difficulty concentrating, tiredness, memory problem.	Antiepileptics (levetiracetam, lamotrigine, valproic acid, controlled- release carbamazepine)	Of the Lack of the 438 patients included, 91 (21%) of them had a Liverpool Adverse event profile of more than 45 indicating a high burden of adverse events.	-Females -Drug resistance -lack of seizures remission -partial epilepsy	Dose reduction Change to another anti-epileptic
Lahon *et al* [32]	Drug related; - neurological - gastrointestinal - hepatic - dermatological - haematological - endocrinological	Antipsychotics, antidepressants and mood stabilisers -serotonin norepinephrine reuptake inhibitors (SNRI), -duloxetine, -Selective serotonin reuptake	There was 119 of adverse drug reactions recorded in 64 cases of the 222 patients included.	unspecified	No life threatening adverse event was reported
Li & Tian [33]	Drug related, allergic and idiosyncratic (Type A and B) drug reactions - Gastrointestinal - Central nervous system related - Respiratory	Oral antibiotics used to treat skin conditions	There were 18 type A ADR that are gastrointestinal-related (12), elevated liver enzymes (2), central nervous system related (2), phototoxicity (1), and amenorrhea (1). There were 10 type B ADR consisting of urticaria (8), exanthema (1), and respiratory related (1). There were 4 ADR in NCCMERP category D and 24 ADR in the category E	unspecified	Unspecified
Lin *et al* [34]	Drug related	Elderly patients receiving inappropriate medications (PIM); - Amiodarone - Chlorzoxazone - Bisacodyl - Nifedipine - Amitriptyline	The incidence of adverse outcome in patients receiving potentially inappropriate medications was 25.1% compared to 17.5% in patients not receiving PIMs (*P* < 0.001).	Elderly patients prescribed a large number if medications Advanced age	Emergency visits (14.6%). Hospitalization (10.1%), Death (0.4%)
Milligan *et al* [35]	Drug related	Insulin therapy and oral therapy	There were 684 reports related to insulin and 84 incidents related to oral glucose-lowering agents.	-Advanced age - administration/supply (69%) - wrong/unclear dose - wrong Strength (25%), - omission of medicine (17%) -Wrong frequency (12%) -Prescribing error (19%)	no harm one death was reported
Montserrat-Capella *et al* [36]	Drug related, allergic - hallucinations, −gastrointestinal bleeding, −constipation/diarrhoea, -convulsions, electrolyte imbalance, -falls - metabolic alkalosis, -nausea/vomiting and -sexual dysfunction	Only specified in specific case studies where examples of adverse events are identified. (e.g. patient diagnosed with Parkinson treated with pramipexole and carbidopa had side effects such as nausea, vomiting and cramps)	The prevalence of adverse events was 5.2% (95%CI 4.2–6.1%)	-Insufficient knowledge of the disease by physician (14.7%), -Short consultation time not being long enough (8.6%) -Incomplete physical examination of the patient (6.9%), -Inappropriate follow-up interval (5.2%). -Patient complexity (11.9%)	Hospital admission Moderate disability to patients (12%). Serious harm to patients (21.4%)
Rosales et al. (abstract) [37]	Drug related	Biological agent Biological agents - (Etanercept (21.3) - Infliximab (22.4%) - Adalimumab (27.3%) - Rituximab (19.2%) - Other BA [Golimumab, Certolizumab, Abatacept and Tozilizumab])	There were 286 courses of biological agents therapy in 146 patients.	-Infection (50%) -Advanced age -Concomitatnt use of corticosteroids -Presence of co-morbidities	Functional loss Infection Death 2.7% Discontinuation of medication (71% in the first year of treatment)
Schildmeijer *et al* [38]	Drug related, allergic, idiosyncratic - Healthcare-associated infections - Falls - Pressure ulcers - Skin, vessel or tissue harm - Pain - Psychological harm - Other - Neurological harm	Unspecified	356 cases of AEs were identified in home care records. (37.7%; 95% CI 33.0 to 42.8)	-Medical conditions (cardiac arrest, DVT, falls, nutritional, pain, oral health, self-inflicted harm) - Laboratory modules - Medication modules - Continuity and transition modules	Temporary harm that required addition or extended healthcare (69.1%)
Shehab *et al* [39]	Drug related, allergic Allergic 1. diarrhoea, dizziness,	- Penicillins - Cephalosporins - Fluoroquinolones - Sulfonamides and trimethoprim - Macrolides and ketolides - Lincosamides (clindamycin) - Metronidazole - Nitrofurans (nitrofurantoin) - Vacomycin and linezolid - Unspecified and other antibiotics - Two antibiotics from different drug classes	From 6614 cases, systemic antibiotic accounted for 19.3% of the total ED visits for drug-related adverse events. A total of 78.7% of drug-related adverse effects were attributed to allergic reaction and 6.1% led to hospitalization	Allergic reactions	Hospitalisations (6.1%) ED visits (19.3%)
Theitler et al. (Abstract) [40]	Drug related fatigue (55.3%), dizziness (18.4%), tremor (15.8%),	Different treatment of AED - phenytoin, -gabapentin, -levetiracetam -lamotrigine	Adverse drug reactions were reported by 34.9 to 49.1% during various visits.	-Comorbidities	Slower titration dose of Levetiracetam
Tomlin *et al* [41]	Drug related, allergic - Nausea, vomiting - Headaches - Sweating - Sedation/drowsiness - Tremors/shaking - Diarrhoea - Insomnia - Anxiety/increased anxiety - Dizziness - Light-headedness - Skin Rash - Anaphylaxis	- Selective serotonin reuptake inhibitors (SSRIs) -Antibacterials -Analgesics -antihypertensive -lipid modifying agents -skin preparations	37,397 reported cases of allergies, adverse events and other warnings, a total of 7.4% of all patients. Adverse event were reported to be related to antibacterial (47.9%), NSAIDs (10.5%), analgesics(7.8%), antihypertensive medicines, lipid-modifying agents and skin preparations	Inconsistent consultation notes, drug safety signals and other medical warnings.	Treatment changes
Tsang *et al* [42]	Drug related, allergic idiosyncratic	- Amoxycillin -Systemic antibiotics - BCG Vaccine - Penicillins - Anti-rheumatics - Atenolol - Simvastatin - Salicylates	The rate of adverse drug reactions was 1.26 reactions per 1000 consultations.	Patients over 65 years Computerized medical records Iatrogenic	Emergency visits hospital admission
Woods *et al* [43]	Drug related	unspecified	There were 827 medication related adverse events (31.7%) of all adverse events. Preventable medications related adverse events were found to be 13.1% ranging from 3.1 to 23.1%.	medication factors (sound-alike, look alike medications) patient-related lack of education	Hospitalisations Serious permanent injuries Death
Wucherer *et al* [44]	Drug related	-Cholinesterase inhibitors -Anticholinergic drugs -Antidementia drugs	6% of the study participants reported adverse drug event related to a prescribed medication Of the 446 participants, 414 had at least one Drug related problem (92.8%). Adverse events were observed in 27 participants with Drug related problem (6.1%).	-Administration and compliance problems -Drug interactions -Inappropriate drug choice -Total number of drugs taken -Formal diagnosis of a mental or behavioural disorder	• unspecified

## Results

**Fig. 1:**
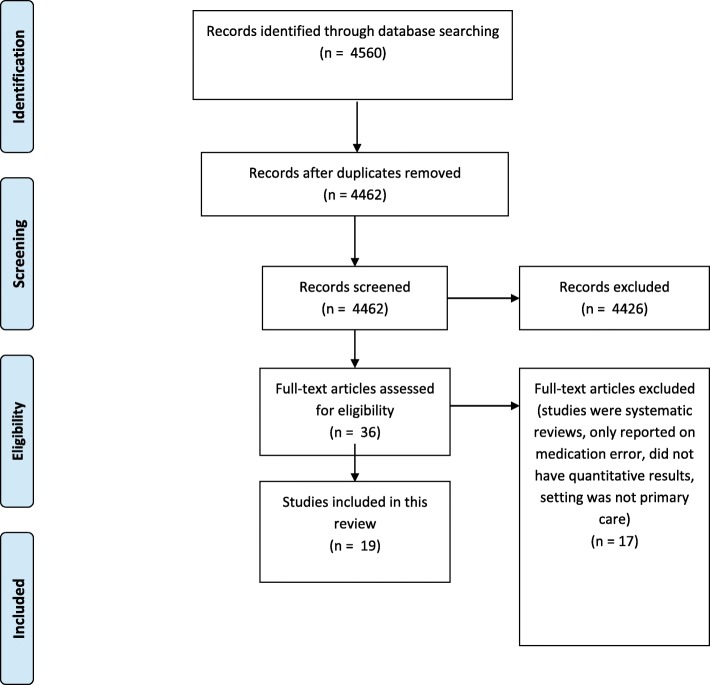
PRISMA flowchart of study selection and inclusion process

The database search yielded a total of 4462 citations after duplicates were removed. The titles and abstracts for these 4462 citations were screened and 4426 had irrelevant titles and abstracts and therefore excluded. The remaining 36 papers were selected for further assessment of the full-text assessment. Of these, 17 were excluded due to having: an irrelevant setting that is not primary care, irrelevant interventions that were only addressing medication errors instead of reporting on drug related adverse events and describing only qualitative aspects of medication safety. The final search yielded a total of 19 citations for inclusion in this review, with two abstracts and 17 full papers [26–44]. A protocol detailing the methodology for the current review was followed [23]. A PRISMA flowchart showing the study selection at each stage is detailed in Fig. 1. Tables 1 and 2 detail the study characteristics and the outcomes.

### Studies characteristics

#### Authors and year of publication/country of origin

The included studies were published between 2003 and 2018. Most of the studies included were undertaken in developed countries such as USA, Germany, Sweden. Details of the studies country of origin are presented in Table 1.

### Study population

The population size for the included studies ranged from 2842 to 33,891,339 patients from across databases searched for this study. The types of participants included elderly residents, cancer patients, epileptic patients, multidrug-resistant TB patients, pediatrics and general adult patients.

## Method

The types of studies included mainly observational cohort studies, retrospective case reviews and health record reviews.

### Context

All studies were conducted in primary care settings. Eleven were set in primary care centers, 12 were set in outpatient clinics, two were set in general practice clinics, one was set in a residential nursing facility, and one was set in home care.

### Type of adverse drug reactions (context)

The types of ADRs are categorized into three groups: drug related, allergic reaction and idiosyncratic reactions. The majority of studies have addressed drug related adverse reactions followed by allergic reactions. Only four studies addressed idiosyncratic reactions [29, 33, 38, 42]. ADRs were classified either by systems (Central nervous systems, cardiovascular events, etc. …) or by adverse reactions (ie. seizures, hearing loss, etc.). The frequency of ADRs reported were not included in all the studies. The most frequent ADR was related fatigue (55%) followed by dizziness (18.4%) and tremor (15.8%) [40]. The body system that was associated with the most ADRs reported was the central nervous system followed by gastrointestinal and cardiovascular systems [26, 28].

### Classes associated with ADRs (context)

A total of nine studies out of the 19 included studies addressed specific classes of medications such as; anti-tuberculosis drugs [29], anti-epileptics [31, 40], antipsychotics, antidepressants and mood stabilisers [32], antibiotics [33, 39], insulin and oral diabetic medications [35], biologicals [37], and anticholinergic drugs including dementia medications [44]. The remainder of the studies covered other classes of medications such as beta blockers, antiplatelets, analgesics, benzodiazepines, musculoskeletal drugs, stimulants, lipid modifying agents, selective serotonin reuptake inhibitors and skin preparations. The classes of drugs that were associated with the highest ADRs reported in the included studies were drugs used for the cardiovascular system (beta-adrenergic blocking agents, diuretics, ACE inhibitors) warfarin, antipsychotic agents and opioids analgesics [26–28].

### ADRs incidences

There was no standardized reporting of the prevalence data in the included studies. Prevalence data varied from simple calculations of the frequency of ADR in the study populations to an estimated number of adverse events per 100 patients, 100 residents’ month, number of reactions per 1000 consultations [26. 28, 42]. Overall, the incidence of ADR reported in the studies ranged between 6% and up to 80% in some cases [29, 44].

### Causes of ADRs reported

The causes of ADR varied between the studies. However, the majority of the authors cited patient factors as the cause of ADRs such as advanced age, lack of patients’ education and patients’ comorbidities [4, 26, 28–31, 34–38, 40, 42].

Some studies mentioned drug related causes such as prescribers’ errors, inconsistent consultation notes, incomplete physical examination, inappropriate follow up and monitoring errors as causes of ADRs [26, 27, 35, 36, 38, 41, 44].

Drug specific causes such as drug administration, dispensing errors, drug interactions and look alike medications were also mentioned by three studies [27, 30, 35, 43].Allergic reactions were cited as the cause of ADRs in one study by Shehab et al. [39] Iatrogenic causes was also cited by one study amongst other causes [42]. Two studies did not specify any causes for the reported ADR [32, 33].

### Consequences of ADRs

The consequences of the ADRs reported in the included studies ranged from medication cessation to death in some cases. Hospitalizations were reported in seven studies [28, 34, 36, 38, 39, 42, 43]. Death was reported in six studies [26, 27, 34, 35, 37, 43].

## Discussion

This scoping review identified that the most causes of ADRs were drug related and due to allergies. Idiosyncratic adverse reactions were not very commonly reported in the literature. This is mainly because it is hard to predict and these reactions are not associated with drug doses or routes of administration [45]. The most common ADRs reported in the studies included in this review were those that are associated with the central nervous system, gastrointestinal system and cardiovascular system. Several classes of medications were reported to be associated with ADRs.

The prevalence of ADRs varied significantly between the studies, reasons for this variation include study designs, characteristics of participants and setting of the study and study length. These results are consistent with a similar review of observational studies [46]. Studies addressing children are also underrepresented in this review. We only found one study that met our inclusion criteria where the authors investigated the rates and types of ADRs in a pediatric ambulatory setting [30].

The causes of ADRs in this review were found to be multifactorial. These included: patient related factors such as co-morbidities, drug interactions, older age, provider characteristics such as monitoring errors, administration errors, incorrect drug selection and drug specific such as allergies or idiosyncratic reactions. Therefore, it is reasonable to predict their occurrences in primary care settings. This is in line with other findings from similar reviews [47].

Hospitalization and mortality were reported in less than half the studies included. Hospitalizations due to ADRs ranged between 6 to 14% which is comparable to other systematic reviews [48–50]. Mortality rates ranged between 0.4 to 2.7% in the studies included in this review. Under reporting of adverse events have been cited in the literature [51]. This may have been due to several factors including barriers to reporting within each organization, clinicians’ reluctance to report to avoid punishment or blame [52]. Other barriers could be lack of knowledge about adverse events and whether they are related to the actual condition or the medications [51, 52]. Complacency and other personal factors related to clinicians such as fear of being ridiculed of reporting merely suspected ADRs and fatigue were also reported [53].

Health care professionals are encouraged to be aware of the most commonly classes of drugs associated with ADRs such as cardiovascular drugs, antipsychotics and opioids as found in these studies. Targeted educational interventions to address underreporting of ADRs is essential to improve public health safety. There are many reasons for underreporting ADRs especially in children is paramount to improve patient safety. Our review highlighted the limited number of studies reporting ADRs in children.

Personal medicine is the approach where health professionals tailor specific treatments for individual patients to optimize outcome and reducing ADRs. As today’s society moves towards personalized medicine, by understanding the causes and nature of ADRs, healthcare providers can extend the benefits and limit adversity on a personal level. By understanding the population and the groups of medications that are particularly susceptible to ADRs, health professionals can make better medication selections and improved dosing for the specific populations [54]. Extensions into research of pharmacogenomics will also improve the understanding of ADRs. Understanding the impact of genetics on drug effects have the potential to predict ADRs.

### Limitations of the review

This review has a few limitations. There was also limited data from the included studies in regard to the ADRs and classes of medications associated with them. Furthermore, most of the studies were undertaken in developed countries. Applying these results to other countries might not be relevant due to the various systems in reporting ADRs. This is in addition to the limitations in the included studies such as small sample sizes, heterogeneous populations, variations in outcome measures.

## Conclusion

This scoping review identified that the most causes of ADRs were drug related and due to allergies. Idiosyncratic adverse reactions were not very commonly reported in the literature.

This is mainly because it is hard to predict and these reactions are not associated with drug doses or routes of administration. The most common ADRs reported in the studies included in this review were those that are associated with the central nervous system, gastrointestinal system and cardiovascular system. Several classes of medications were reported to be associated with ADRs.

## Data Availability

Not applicable.

## Abbreviations

ADRs: Adverse drug reactions
ACE inhibitors: Angiotensin converting enzyme inhibitors
PRISMA: Preferred Reporting Items for Systematic Reviews and Meta-Analyses
TB: tuberculosis
